# Can statins lessen the burden of virus mediated cancers?

**DOI:** 10.1186/s13027-022-00460-0

**Published:** 2022-09-04

**Authors:** Eva H. Clark, Sarah T. Ahmed, Elaine Chang, Elizabeth Y. Chiao, Donna L. White

**Affiliations:** 1 Section of Infectious Diseases, Department of Medicine, Baylor College of Medicine, Houston, TX USA; 2 Center for Innovation, Quality, Effectiveness and Safety (IQuESt), Michael E. DeBakey VA Medical Center, Houston, TX USA; 3 Section of Health Services Research, Department of Medicine, Baylor College of Medicine, Houston, TX USA; 4 Departments of Epidemiology and General Oncology, MD Anderson Cancer Center, Houston, TX USA; 5 Section of Gastroenterology and Hepatology, Baylor College of Medicine, Houston, TX USA; 6 Section of Pediatric Tropical Medicine, Baylor College of Medicin, Feigin Building Suite 550, Houston, TX 77030 USA

**Keywords:** Oncogenic virus, Cancer, Statin, Virus-mediated malignancies, HBV, HCV, HPV, EBV, KSHV, HHV8

## Abstract

**Background:**

Oncogenic viruses, including hepatitis B virus (HBV), hepatitis C virus (HCV), human papillomavirus (HPV), Epstein Barr virus (EBV), and Kaposi Sarcoma Herpes virus (KSHV) contribute to a significant proportion of the world’s cancers. Given the sizeable burden of virus mediated cancers, development of strategies to prevent and/or treat these cancers is critical. While large population studies suggest that treatment with hydroxymethylglutaryl-CoA reductase inhibitors, commonly known as statins, may reduce the risk of many cancer types including HBV/HCV related hepatocellular carcinoma, few studies have specifically evaluated the impact of statin use in populations at risk for other types of virus mediated cancers.

**Main body:**

Studies of populations with HBV and HCV suggest a protective, dose-dependent effect of statins on hepatocellular carcinoma risk and support the theory that statins may offer clinical benefit if used as chemoprophylactic agents to reduce liver cancer incidence. However, no population level data exists describing the impact of statins on populations with other oncogenic viral infections, such as HPV, EBV, and KSHV.

**Conclusion:**

Further study of statin use in diverse, global populations with or at high risk for oncogenic viral infections is essential to determine the impact of statin therapy on virus mediated cancer risk.

## Background

Cancers caused by infections represented more than 13% of the global cancer burden in 2018, including more than 1.2 million cancers caused by viruses [1]. Of these, hepatitis B and C viruses (HBV and HCV) contributed to approximately 520,000 cancers, and human papilloma viruses (HPV) caused approximately 690,000 cancers. The remaining virus mediated cancers were due to viruses including Epstein-Barr virus (EBV), Kaposi Sarcoma Herpes virus (KSHV, also known as human herpesvirus type 8 [HHV8]), and human T-cell lymphotropic virus type-1 (HTLV-1). Two-thirds of these infection-attributable cancers occur in low- and middle-income countries [2]. Though vaccines to prevent HBV and HPV have been available for more than a decade and antiviral therapy has made HCV essentially a curable disease [3], significant obstacles continue to prevent effective implementation of such tools, especially in the countries with the largest burdens of virus mediated cancers.

Hydroxymethylglutaryl-CoA (HMG-CoA) reductase inhibitors, commonly referred to as “statins,” potentially have a significant impact on the burden of virus mediated cancers and should be investigated as well-tolerated agents that may reduce the risk of developing such cancers. The objectives of this article are to (1) summarize available epidemiologic data describing the impact of statin therapy on cancers related to specific oncogenic viral infections (specifically, HBV, HCV, and HPV; no published studies on this topic exist for EBV, KSHV, HTLV-1, or Merkel cell polyomavirus [MCPyV]) and (2) identify gaps for future investigation of the impact of statins on virus-mediated cancers.

## Main text

### Role of oncogenic virus infections on cancer development

While many viruses can cause chronic inflammation and immune dysregulation, only seven oncogenic viruses are known to cause human malignancies [4]. These seven diverse viruses share several characteristics that permit and facilitate carcinogenesis, including deactivation of tumor suppressor pathways, deregulation of host signaling pathways, blunting of the host response to DNA damage, and inhibition of antitumor immune surveillance and response [4–6]. However, while viral infection is necessary to cause certain cancers, it is not sufficient [5]. Additional factors, ranging from environmental exposures to host genetic and immunologic susceptibility, are likely necessary for malignant transformation of the host-cell genome [7]. Given the long latency period between viral infection and cancer development (often decades [7]), it may be possible to prevent or slow the development of virus mediated cancers by reducing an individual’s exposure to any or all of the processes described above.

### Impact of statins on cancer incidence and prognosis

As oncogenic virus infections are often asymptomatic and therefore can persist for years before detection, reduction or prevention of the disease caused by oncogenic virus infection via empiric treatment of at-risk populations with an oral medication that is safe and well-tolerated would be an ideal solution. Statins could represent such a medication. Statins were developed in the 1970s–80s to prevent arterial disease via reduction of total and low density lipoprotein (LDL) cholesterol levels [8]. Now they are widely prescribed for their therapeutic and primary and secondary preventive effects in cardiovascular disease [9–12].

The same characteristics that make statins attractive as a tool to prevent cardiovascular disease have led to interest in them as agents that may reduce cancer risk. Pre-clinical data suggest that statins have pleiotropic anti-inflammatory effects [13–19] and contribute to activation of molecular cascades essential to survival of cancer cells [20, 21]. Specifically regarding their potential to reduce cancers mediated by oncogenic viruses, some evidence suggests that statins inhibit HCV [22, 23] replication, downregulate HBV activity and replication [24], and improve the antiviral activity of several HCV therapies [25].

Numerous publications have evaluated the relationship between statins and various types of cancer [26, 27]. Though robust evidence for a positive relationship between statin use and reduced cancer risk has not been conclusively shown for all cancer types, several large, diverse, population-based epidemiologic studies note a lower cancer incidence and/or mortality in populations treated with statins, including for cancers such as colorectal [28–30], prostate [31–33], gastric [34], pancreatic [35, 36], liver [37–40], breast and cervical [41, 42], endometrial and ovarian [43, 44], and lymphoma [45, 46].

### Statins and liver cancer caused by HBV and HCV

Statins have been considered specifically for liver cancer prevention as they undergo hepatic first-pass metabolism and sequester in the liver. Available data evaluating the question of whether statins directly reduce liver cancer incidence are limited by the fact that liver cancer is a rare cancer that takes years to develop and by the paucity of studies able to stratify their data by the various etiologies of liver cancer (e.g., viruses, aflatoxin exposure, alcohol exposure, and non-alcoholic fatty liver disease), as likely etiologies are regional and differ significantly among the populations studied to date. Even so, several meta-analyses show that statin use is significantly associated with a reduced risk of hepatocellular carcinoma (HCC) of any etiology [47, 48]. Specifically, statin use may lead to an improved virologic response to treatment and reduced risk of cirrhosis and HCC in populations with chronic HBV and HCV infections [49, 50]. Table 1 describes seven cohort studies evaluating the incidence of HCC in populations with chronic HBV or HCV infections. Despite significant heterogeneity in reporting methods, all seven studies found that the risk of incident HCC was significantly reduced in most types of statin user groups compared to non-users (Tables 2A [HBV] and Table 2B [HCV]). The hazard ratios describing HCC risk reported by the these studies are similar to effect ratios reported by previous systematic reviews and meta-analyses that evaluated statin use on risk of HCC of any cause (e.g., in [47], risk ratio = 0.60 [95% CI 0.53–0.69]) and on risk of HCC in populations with HCV (e.g., in [49], relative risk = 0.45 [95% CI 0.36–0.57]).

**Table 1:** Characteristics of seven studies evaluating statins and incidence of liver cancer in people with hepatitis B or C virus infection

Study	Years of study	Country	Study design	Number of centers	Age (years)	No. total population initially evaluated	No. HBV included in analysis	No. HCV included in analysis	No. male	% male	Age (mean or median)	No. statin users	% statin user	Statins evaluated	Primary outcome
Goh [52]	2008–2012	South Korea	Retrospective cohort	Single center	≥ 18	12,950	7713	N/A	5106^c^	66%^c^	50^c^	713	9	A,F,P,Pi,R,S	HCC incidence
Hsiang [54]	2000–2012	China	Retrospective landmark analysis, followed by propensity weighting	Nationwide cohort	≥ 18	77,021	53,513	N/A	14,145^c^	26%^c^	38^c^	1176	2	A,F,R,S	HCC incidence
Simon [55]	2001–2014	US	Retrospective cohort	Nationwide cohort	N/A	47,549	N/A	9135	8743^c^	96%^c^	53	4165	46	A,C,F,L,P,S	HCC incidence
Simon [56]*	2005–2013	Sweden	Prospective propensity score-matched cohort	Nationwide cohort	≥ 18	16,668	3906	12,762	1992^c^	51%^c^ (HBV), 62%^c^ (HCV)	35^c^ (HBV), 39^c^ (HCV)	1953 (HBV), 6381 (HCV)	50	A,P,R,S	HCC incidence
Tsan [50]	1997–2008	Taiwan	Retrospective cohort	Nationwide cohort	> 18	33,413	33,413	N/A	19,442	58%	36	2785	8	A,F,L,P,R,S	HCC incidence
Tsan [57]	1999–2010	Taiwan	Retrospective cohort	Nationwide cohort	> 18	260,864	N/A	260,864	128,263	49%	50	35,023	13	A,F,L,P,R,S	HCC incidence
Yang [58]**	1996–2005	China	Retrospective cohort	Nationwide cohort	N/A	1319	204	N/A	141	69%	52	148	28	Not specified	HCC incidence

*Note that this publication is included in both parts of Table 2 (its HBV data in Table 2A and it's HCV data in Table 2B)

**This cohort includes only persons with diabetes

^c^Value calculated from available study data

*HBV* hepatitis B virus, *HCV* hepatitis C virus, *HCC* hepatocellular carcinoma, *US* United States, *A* = atorvastatin, *C* cerivastatin, *F* fluvastatin, *L* lovastatin, *P* pravastatin, *Pi* pitavastatin, *R* rosuvastatin, *S* simvastatin

**Table 2:** (A) HCC Incidence and hazard ratios in studies evaluating people with HBV and (B) HCC incidence and hazard ratios in studies evaluating people with HCV

All						Statin users										Statin non-users			
Study	Follow up period	No. HCC cases	HCC incidence (%)	HCC incidence rate (per 100,000 py)	Model adjustment(s)	No. HCC cases	HCC incidence (%)	HCC incidence rate (per 100,000 py)	(a) HR and 95% CI							No. HCC cases	HCC incidence (%)	HCC incidence rate (per 100,000 py)	HR and 95% CI
									All	1st cDDD group	2nd cDDD group	3rd cDDD group	4th cDDD group	LS group	HS group				
**(A)**																			
Goh [52]	7.2 years	702	9.1	N/A	Age, sex, cirrhosis, DM, HTN, HBV DNA level, ALT level, total cholesterol, antiviral/anti-platelet medications	30	3.3	N/A	0.36, 0.19–0.68	0.63, 0.31–1.29 [28–365 cDDD]	0.51, 0.21–1.25 [366–730 cDDD]	0.32, 0.07–1.36 [731–1095 cDDD]	0.17, 0.06–0.48 [> 1095 cDDD]	0.35, 0.16–0.78	0.39, 0.15–1.05	672	7.9	N/A	Ref
Hsiang [54]	393,154 py	1298	2.43^c^	520	Propensity score	36	3.1^c^	1240	0.68, 0.48–0.97							1262	2.4^c^	510	Ref
Simon [56]*	8 years	136^c^	3.5^c^	289^c^	Propensity score	54^c^	2.8^c^	209^c^	N/A					0.58, 0.48–0.78	0.94, 0.84–1.14	82^c^	4.2^c^	386^c^	Ref
Tsan [50]	328,946 py	1021	3.1^c^	310	Age, sex, income, urbanization, diabetes, cirrhosis	58^c^	2.1^c^	211	0.47, 0.36–0.61	0.66, 0.44–0.99 [28–90 cDDD]	0.41, 0.27–0.61 [91–365 cDDD]	0.34, 0.18–0.67 [> 365 cDDD]		0.44, 0.33–0.59	0.51, 0.31–0.85	963	3.1^c^	320	Ref
Yang [58]**	5782 py	23	1.7	398	Age sex, tobacco, alcohol, BMI, cirrhosis	1	2.9	N/A	13.64, 0.84–221.23							13	8.9	N/A	57.22, 6.95–470.96
**(B)**																			
Simon [55]	97.9 mos (statin users); 81.6 mos (nonusers)	239	2.6	N/A	Baseline FIB-4	73	1.8	300 (28–89 cDDD), 200 (90–180 cDDD), 215 (> 180 cDDD)	0.53, 0.36–0.72	0.85, 0.47–1.53 [28–89 cDDD]	0.48, 0.27–0.88 [90–180 cDDD]	0.51, 0.36–0.72 [> 180 cDDD]				160	3.2	465	Ref
Simon [56]*	8 years	480	3.8^c^	296^c^	Propensity score	211	3.3^c^	232^c^	N/A					0.54, 0.45–0.83	0.96, 0.87–1.10	269	4.2^c^	377^c^	Ref
Tsan [57]	2,792,017 py	27,883	10.7	999	Age, sex, urbanization, income, cirrhosis, DM	1378	3.9^c^	341	0.53, 0.49–0.58	0.66, 0.59–0.74 [28–89 cDDD]	0.47, 0.40–0.56 [90–180 cDDD]	0.33, 0.25–0.42 [> 180 cDDD]				26,505	11.7^c^	1110	Ref

(a) *HR* hazard ratio (adjusted HR reported if available), *cDDD* cumulative defined daily statin doses, *DM* diabetes, *FIB-4* fibrosis-4 score, *HBV* hepatitis B virus, *HCC* hepatocellular carcinoma, *HTN* hypertension, *HS* hydrophilic statin users, *LS* lipophilic statin users, *py* person years, *Ref* reference

*Note that this publication is included in both tables (its HBV data in Table 2A and it's HCV data in Table 2B)

**This cohort includes only persons with diabetes

^c^Value calculated from available study data

Dose and duration of statin therapy appear to significantly impact risk of HCC, as shown in a 2016 meta-analysis of 25 studies (including 12 cohort studies, 10 case–control studies, and three post-hoc analyses of randomized controlled trials) providing the risk ratio (RR) for statin use and primary liver cancer (of any cause/type) risk. This 2016 meta-analysis not only found that statin use was significantly associated with a reduced risk of primary liver cancer (RR = 0.60, 95% CI = 0.53–0.69), but also that the RR for every additional 50 cumulative defined daily doses per year was 0.87 (95% CI = 0.83–0.91) [47]. The same study suggested an additional benefit of statin use in high-risk populations, such as those with HBV or HCV infection; specifically, they estimated a RR of 0.50 (95% CI = 0.36–0.69) for those with HBV infection and 0.53 (95% CI = 0.49–0.57) for those with HCV infection. In 2017, a large propensity-matched population study of patients with liver cirrhosis associated statin use with decreased risk of decompensation, mortality, and HCC in a dose-dependent manner, particularly in those with chronic HBV/HCV infections [51]. Collectively, this is suggestive supporting evidence that higher statin doses and/or longer statin durations of use may prevent or delay liver cancer development in people with chronic HBV/HCV infections.

Further, the question of whether lipophilic or hydrophilic statin use has a greater impact on HCC risk in HBV or HCV infected individuals has been specifically addressed by three studies. First, in Tsan et al.’s 2012 large retrospective cohort study of people with HBV infections, both lipophilic and hydrophilic statin users had reduced risk of HCC development compared to statin non-users (adjusted HR was 0.44 [95% CI 0.33–0.59] for lipophilic statin users and 0.51 [0.31–0.85] for hydrophilic statin users) [50]. In contrast, Simon et al.’s 2019 prospective propensity-matched study found that 10-year HCC risk was significantly lower among lipophilic statin users compared to stain non-users but this risk reduction was not seen for hydrophilic statin users compared to statin non-users (for people with HBV, adjusted HR was 0.58 [95% CI 0.48–0.78] for lipophilic statin users and 0.94 [95% CI 0.84–1.14] for hydrophilic statin users; for people with HCV, adjusted HR was 0.54 [0.45–0.83] for lipophilic statin users and 0.96 [0.87–1.10] for hydrophilic statin users). Goh et al. reported similar findings in their 2020 single center study [52].

### Statins and cancers caused by HPV

In contrast to HBV/HCV, few epidemiologic studies describe the impact of statin use on HPV-, EBV-, or KSHV-mediated cancers. For HPV, one retrospective cancer database study of 1638 individuals with head and neck cancers found a statistically significant inverse association between ever using a statin and death from HPV + head and neck cancers (HPV-positive HR = 0.41, 95% CI 0.21–0.84; HPV-negative HR = 1.04, 95% CI 0.71–1.51) [53].

## Conclusions and future directions

Available data suggest a protective, dose-dependent effect of statins on HCC risk in populations with chronic HBV and/or HCV infections. Thus, statins may show clinical benefit if used as chemoprophylactic agents to reduce the public health burden of HCC, and randomized clinical trials evaluating the effectiveness of statins in HCC outcomes both with other therapies (NCT03275376) and to prevent recurrent HCC (NCT03024684) are ongoing. Trials being planned should include comparisons of lipophilic or hydrophilic statins in their designs.

The promise of statins in preventing HCC warrants extrapolation of epidemiologic and clinical study of statin therapy to populations at risk for other oncogenic viral infections, particularly HPV, EBV, and KSHV. Importantly, despite the much larger global burden of cervical cancer, the only published study describing the impact of statins on HPV is in HPV-related head and neck cancers, which are predominantly diagnosed in men in high income countries. Future studies must rectify this disparity by evaluating the impact of statins on cervical cancer, particularly in low- and middle-income countries where cervical cancer rates are higher due to poor access to HPV vaccines and surveillance programs, but also in high income countries where cervical cancer remains an important cause of cancer morbidity and mortality despite robust HPV prevention programs. Statin therapy might prevent or slow the development of cervical cancer if initiated immediately after detection of high-risk HPV subtypes or cervical dysplasia in women undergoing cervical cancer screening. Though pre-clinical data suggest that statin therapy may ameliorate EBV-related cancers, no epidemiologic studies have evaluated statins in prevention or as an adjunctive therapy in at risk populations. Such studies are needed to determine whether statin intervention trials are warranted to prevent EBV-related cancers. Though KSHV afflicts a large proportion of the population, particularly in sub-Saharan Africa, no investigations into the ability of statin therapy to limit the development of KSHV-associated cancers exist; study of the relationship between KSHV and statins should be initiated via in vitro and animal model studies. Finally, HIV hugely impacts risk of morbidity and mortality in those co-infected with the oncogenic viruses covered in this Review, and several anti-retroviral therapies in widespread use are known to contribute to dyslipidemia. Future research should include investigation of statins as agents to prevent virus mediated cancers in people with HIV, as these individuals may have different outcomes or require different statin doses.

The studies outlined in this article provide preliminary evidence that statins may induce mechanisms that slow virus-mediated cancer development, which should be further investigated in epidemiologic studies of populations at risk for HPV, EBV, and KSHV infection. Randomized trials and large community-based prevention trials prospectively evaluating use of specific statins in populations with or at high risk for oncogenic viral infections will be needed to further explore the potential benefit of statins in decreasing virus mediated cancer risk.

## Data Availability

All data generated or analyzed during this study are included in this published article.

## Abbreviations

EBV: Epstein-Barr virus
HBV: Hepatitis B virus
HCC: Hepatocellular carcinoma
HCV: Hepatitis C virus
HPV: Human papilloma viruses
KSHV: Kaposi Sarcoma Herpes virus
LDL: Low density lipoprotein
NPC: Nasopharyngeal carcinoma
